# Effects of Asian cultural values on parenting style and young children’s perceived competence: A cross-sectional study

**DOI:** 10.3389/fpsyg.2022.905093

**Published:** 2022-10-17

**Authors:** Eunice Pui-Yu Yim

**Affiliations:** School of Education and Languages, Hong Kong Metropolitan University, Kowloon, Hong Kong SAR, China

**Keywords:** parenting style, cultural values, perceived competence, child development, immigrant

## Abstract

Authoritarian parenting has long been associated with Western individualism and improved child development. This study examined the relationship between cultural values, parenting styles, and children’s perceived competence in Hong Kong. A total of 48 parents from local Chinese families, 49 parents from South Asian families, and 105 children (24 local Chinese and 81 South Asian) aged 5–6 years participated in the study. Self-report questionnaires on adherence to Asian cultural values and parenting style were administered to parents. The Pictorial Survey on Children’s Perceived Competence was administered to children by trained research assistants. The results contradicted two long-standing assumptions on Asian cultural values and parenting styles. First, higher adherence to Asian cultural values increased the likelihood of having an authoritarian parenting style. Second, authoritative parenting practices were more likely be associated with improved social–emotional competence in children. Multiple regression analyzes revealed a strong positive correlation between Asian cultural values and authoritative parenting style (*R*^2^ = 0.597). There was no association between parenting style and the development of competence in young children. However, a positive correlation was found between Asian cultural values and young children’s perceived competence. This study showed that components of collectivism and humility in Asian cultural values could have functional values that are essential for developing competencies in South Asian young children but not in local Chinese young children. This study discussed the implications of cultural values sin the terms of contextualization, functional relevance of cultural values for ethnic minorities, and ideal parenting practices.

## Introduction

In recent decades, the ethnic diversity in Hong Kong has expanded (Fleming, 2019). In 2016, Hong Kong had a population of approximately 7.34 million, including 8% of the population that is non-Chinese compared to the 6% of non-Chinese in 2011 (Census and Statistics Department, 2011, 2016). South Asian ethnic groups, such as Indonesians, Nepalese, Pakistanis, and Filipinos, comprised 71% of the non-ethnic Chinese population in Hong Kong (Census and Statistics Department, 2016). South Asians and local Hong Kong citizens are categorized as Asians and assumed to have a collectivist cultural orientation (Fleming, 2019). However, there are variations in the different cultural sub-groups across the broader Asian cultures, which are reflected in every aspect of life, including parenting practices.

Bronfenbrenner’s (1979) ecological systems model of development proposes that an individual is nested within interrelated social systems at different levels, ranging from the micro level, (referring to the cultural context in the home) to the macro level (the broader socio-cultural context). Childrearing is inevitably affected by the occurrences within these social levels and vice versa (Bronfenbrenner, 1986). Parenting comprises various practices that reflect the cultural values within a child’s home that could differ from the broader local culture in which they live (Bornstein, 2012; Hosken et al., 2019).

Acculturation theory contends that the smooth transition across contexts and positive acculturation with the host culture is required among immigrants (Berry, 1980; Barker and Cornwell, 2019). The smooth transition across contexts is indicative of having knowledge of the social expectations of the involved contexts and behaving in accordance to these expectations (Schwartz et al., 2010; Li, 2013). Given this, an individual may engage himself or herself in forced adaptation, not yet positive acculturation, in order to obtain smooth transitions. Acculturation theory suggests that forced adaptation and positive acculturation represent two ends of a continuum (Berry, 1980; Barker, 2015). New immigrants frequently engage in “forced” adaptation wherein they conform to local cultural values at the early stage of the adaptation process where as positive acculturation refers when individuals can achieve a balance between host (macro-level) and home (micro-level) cultural values in which they exercise these values appropriately and adaptively without devaluing either of the cultural values (Schwartz et al., 2010). In other words, positive acculturated individuals can exercise more than one set of cultural values over time and within or across contexts in daily living smoothly and competently. Considering this, exploring the dynamics between Asian cultural values on parenting behaviors would provide insights into the heterogeneity of Asian cultural values in Asian sub-cultural groups and their relationship to parental behaviors, which in turn influence children’s perceived competence.

Cultural studies have identified six key elements typically associated with an authoritarian parenting style that most Asian ethnic groups having a collectivist cultural orientation share (Kim et al., 1999; Ng and Wang., 2019). These elements are: (1) conformity to social norms (i.e., recognition and adherence to social expectations, norms, and practices); (2) family recognition through achievements (i.e., a reflection of an individual’s achievements and family status); (3) emotional self-control (i.e., behaving appropriately rather than acting on how one is feeling); (4) collectivism (i.e., placing group welfare before individual welfare); (5) humility (i.e., being humble without boasting about one’s achievement); and (6) filial piety (i.e., being respectful toward one’s living parents, ascertaining the rules of culturally defined intergenerational relationships, and placing familial needs before individual interests). Among the four parenting styles proposed by Diana Baumrind (1978), the authoritarian parenting style is characterized by high control and low warmth in which parents employ strict discipline, are insensitive to the child’s emotional needs (Ang and Goh, 2006). Parenting behaviors of this style include expectations of obedience and compliance to parental demands with less responsiveness toward their children’s needs (Rudy and Grusec, 2006). Existing research suggests that parents in Asian societies generally demand high obedience levels from their children and interact with family and community members through rules and orders based on social hierarchy (Rudy and Grusec, 2006; Dwairy and Achoui, 2010). On the other hand, while permissive parenting style cares about their children’s needs but have difficulty setting limits in children’s disciplines, the authoritative parenting style is characterized by high control and high warmth with high parental affection and responsivity to children’s needs as well as high expectations with increased independence and self-control (Rudy and Grusec, 2006). These characteristics are generally reflected individualism-oriented culture which is characterized by open discussions and interpersonal equality dominating the Western culture (Rudy and Grusec, 2006). It is considered the ideal parenting style to raise competent children, whereas the authoritarian style is more likely to raise less competent children (Sorkhabi, 2005; Xu et al., 2005; Chen-Bouck et al., 2019).

Chao (1994) explained the discrepancies between Western and Chinese cultures in their understanding of “control” and “love.” Particularly, the meaning of “guan” (to control) in the Chinese culture includes the meaning of responsiveness to children’s needs has been misinterpreted by non-ethnic Chinese cultural groups. “Guan” means “to govern” but also means “to care for and to love.” Components of love and responsiveness are embedded in the notion of “control” in Chinese parenting. The underlying meaning of “control” for them is to protect children from harm and help them succeed (Ang and Goh, 2006; Mousavi and Juhari, 2019). Therefore, Chinese parents, view “control” as an act of parental love, care, involvement, concern, and support for children’s needs rather than the negative connotations suggested in existing family studies (Chao, 1994; Sorkhabi, 2005; Xu et al., 2005).

Other studies on the association between parenting style and children’s positive outcomes highlighted the need to contextualize cultural values with consideration of children’s developmental stage, engaged cultural contexts and meanings to the families and their children (Alegre, 2011; Huang and Gove, 2015; Hung, 2018; Olla et al., 2018). Studies demonstrated that Chinese youth living in authoritarian families performed better in school than those in authoritative families (Alegre, 2011; Hung, 2018). Other studies have investigated the association between authoritarian parenting in Asian families and their children’s development (Huang and Gove, 2015; Hung, 2018; Olla et al., 2018). These studies suggested that parents had their own values and expectations about how they, as parents, should behave to raise competent children. Many of these parents described competence in terms of academic achievement (Huang and Gove, 2015; Hung, 2018; Olla et al., 2018). On the other hand, studies on authoritative parenting behaviors found that these behaviors significantly contributed to improved school grades for non-Chinese, European-Americans youths, and school-aged children more than the first-generation Chinese youth and school-aged children (Pong et al., 2010; Antony-Newman, 2019). These findings suggested that the characteristics of authoritative parenting may not exert the same effects on families from different cultural backgrounds. Adolescents and young children are at different developmental stages. Adolescents experience more academic pressure for school achievement than young children, while young children experience challenges related to socio-emotional development such as acceptance from others, including parents, caregivers, and peers. Besides focusing on academic achievements and decontextualizing the interpretations of Asian cultural values, the existing studies focus on the influence of Asian cultural values on parenting while targeting Asian families with adolescents in different countries instead of young children from different sub-cultural group in the same geographic region (Chao, 2001; Pong et al., 2005; Shoshani and Steinmetz, 2014) and measuring developmental outcomes in adolescents in terms of academic achievement (Chao and Kaeochinda, 2010; Lin et al., 2021). Thus, assessments of young children’s development should focus on social–emotional development such as perceived self-competence in daily life which is affected by the children’s involved social systems (Brock et al., 1998; Huber et al., 2019).

Bronfenbrenner’s ecological and bioecological model of development provides a theoretical framework that describes the interconnectedness across five major social systems and their influence on personal development from an individual, societal, and organizational level (Bronfenbrenner, 1986; Bronfenbrenner and Morris, 1998). These five systems include: (1) the *microsystem,* which is the context that is closest to an individual’s life with the most direct interactions (e.g., family, school, religious institutions, neighborhood, and peers); (2) the *mesosystem,* referring to the interconnections between the different elements in an individual’s microsystems, (e.g., interactions between the family and school or children’s peers and the family); (3) the *exosystem,* representing the links between social settings in which an individual is not actively involved (e.g., a child’s experience at home may be influenced by parental experiences at work, subsequently affecting parent–child interactions); (4) the *macrosystem*, comprising the overarching societal culture (e.g., socioeconomic status, ethnicity, and laws and rules binding individuals); and (5) the *chronosystem*, which refers to how the person and environments change over time (Christensen, 2016; Xia et al., 2020). According to this model, instead of living within five separate social systems, individuals are nested within the five social systems that are embedded within one another. The five social systems are embedded such that individuals are affected by the progressively complex reciprocal interactions between an individual and his or her environment regularly over extended periods (Xia et al., 2020). The model was later enhanced by introducing Process–Person–Context–Time Model (PPCT) to highlight on the important role of time and developmental needs across the life span on an individual development (Bronfenbrenner, 1992; Xia et al., 2020).

Proximal processes (*Process*) are the reciprocal interactions between a developing human being and one or more of the persons, objects, and symbols in his or her immediate environment (Bronfenbrenner, 1992). These reciprocal interactions are expected to become “progressively more complex” with the expanded social network and more complicated life events (Bronfenbrenner and Morris, 1998). *Person* characteristics include personality traits that can foster or sustain proximal processes, or interfere with or prevent their occurrence. The sustainability of the proximal process can be further supported by those biological, mental, or experiential resources, such as physical attributes, and reading abilities, that individuals bring to the development process. Contextual demands that may pose barriers to the development and proximal processes in the individuals vary from time to time, such as the difficulties of tasks and whether the demands posed on the individual are beyond the individual’s capabilities and resources available at the time. *Context* refers to the socio-cultural environment, including digital world where the individual engages. Johnson and Puplampu (2008) proposed an ecological techno-subsystem highlighting the significant impacts of the availability of technologies in an individual’s microsystem that could bring socio-cultural influences from virtual contexts in which the individual is not currently physically engaged. In other words, individuals are exposed to multiple cultures and norms rather than being confined to a single culture or norm (Johnson and Puplampu, 2008; Schwartz et al., 2010). Regarding the last component of *Time*, the model suggested that development and reciprocal interaction can occur within a shorter period during a proximal process (microtime) or span over a longer period of days to months (mesotime) or decades within and across generations (macrotime; Bronfenbrenner, 1986; Bronfenbrenner and Morris, 1998).

While the refined bioecological model with the PCCT model and ecological techno-subsystem provided a general overview of how different factors could synergistically impact human development over extended periods, it fails to explain how an individual is affected by the socio-cultural context it is engaged in at a specific developmental stage. In some stages of development, some factors may exert more influence on the individual’s development than the others. Different competence weighs differently in the different developmental stages. In childhood, the socio-emotional competence is considered more important than academic related competences, which are weighted heavier in school-aged and later stages of development. In this study, young children of 5 years old who are in the early stage of development (*Time*) and they primarily engage themselves in home and school (*Context*). Young children generally spend considerable less amount of time on internet than older children (Hinkley et al., 2012; Fakhouri et al., 2013). The reciprocal interactions between the children (*Person*) and their major caregiver (s) are the major proximal processes (*Process*) to be measured.

Review of the existing research investigating associations between parenting styles and cultural values have the following limitations. First, the existing literature investigating the parenting styles of Asian sub-cultural groups mainly compared families of inter-Asian countries (Ang and Goh, 2006; Chuang et al., 2018). Second, studies investigating the effects of parenting styles on children’s development mainly focus on adolescents or school-aged children than preschoolers as study samples with research emphasis on academic-related skills for school readiness rather than their socio-emotional development (Wong et al., 2018; Xia, 2020). Third, although there are studies on Hong Kong-Chinese and sub-Asian children in Hong Kong, these studies focus on parental expectations and satisfaction instead of the effects of their parenting on the socio-emotional development of young children (Chan et al., 2009; Chan and Li, 2020; Lau and Power, 2020). The limitations in the existing literature suggest a need to explore the differences in parenting practices among Asian sub-cultural groups in within the same geographical region like Hong Kong (Chuang et al., 2018). Hong Kong, previously a British colony, has its Chinese cultural origins that can be discerned along with influence of the Western cultures. Interpretations of Asian cultural values among local Hong Kong residents are not equivalent to those who immigrated to Hong Kong from neighboring Asian countries (Hue and Kennedy, 2014). With an increasing number of South Asian families are migrating to Hong Kong with young children., the intergroup differences between local Chinese and South Asian cultural groups must be investigated to understand how adherence to Asian cultural values contributes toward parenting styles and the influence of parenting style on children’s development within these groups (Yuen, 2016). The study investigates the extent to which these sub-cultural groups adhere to Asian cultural values and how these values were related to their choice of parenting practices which in turn contribute to children’s well-being. This study aimed to investigate (1) the intergroup differences between local Chinese parents and South Asian parents in their adherence to Asian cultural values and their parenting style; (2) the relationship between Asian cultural values and parenting styles; (3) the dynamics between the specific components of Asian cultural values and young children’s perceived competence; and (4) relationship between parenting style and children’s perceived competence.

## Materials and methods

### Participants

Local kindergartens with South Asian families in districts with households of lower socio-economic status were invited to participate in the study through open recruitment. Three kindergartens agreed to participate. Children aged between 5 and 6 years and their parents were recruited from the participating kindergartens. The average age of parents was 33.2 years old, ranging from 21 to 33 years old. One kindergarten school had over 90% South Asian children and 10%, local Chinese children, while the second one had 50% South Asians and 50% local Chinese children, and the third had 90% local Chinese children with 10% South Asian children. The South Asian ethnic groups included Indonesians (33%), Nepalese (11%), Pakistanis (34%), and Filipinos (22%). Due to the limited sample size of South Asian samples, this study used South Asian as the collective term for the South Asian ethnic groups and local Chinese for Hong Kong local ethnic group.

According to the school principals and teachers of participating schools, the South Asian parents had settled in Hong Kong for over 5 years and could read and speak English, with their children being born and raised in Hong Kong. No formal assessment was administered to determine children’s language proficiency in Cantonese. These South Asian children started their full-day schooling in the participating schools when they were 3 years old. After 2 years of schooling in local kindergartens, these children were proficient in Cantonese for daily communication. Major caregivers for the children were invited to participate in the study. All individuals participating voluntarily in the study comprised 97 parents (49 mothers from South Asian group; 48 mothers from local Chinese groups), and 105 children (24 local Chinese, 81 South Asian).

### Materials

This study employed three scales to assess an individual’s adherence to Asian values, parenting style, and children’s perceived competence. These scales were: 1) the Asian Values Scale, 2) the Parenting Practice Questionnaire, and 3) the Pictorial Scale of Perceived Competence and Social Acceptance for Young Children (PSPCSA) Preschool-kindergarten (both the boys and girls versions).

#### Asian values scale

The Asian Values Scale was a self-administered questionnaire developed by Kim et al. (1999) to assess parents’ adherence to Asian cultural values. The scale’s 24 items were rated and scored on a five-point Likert scale from 1 (*strongly disagree*) to 5 (*strongly agree*) and measured an individual’s adherence to Asian cultural values. The six domains included: *conformity to norms* (eight items), *family recognition through achievement* (three items), *emotional self-control* (three items), *collectivism* (three items), *humility* (three items), and *filial piety* (four items). The scale has been used in several countries (including America, Australia, Philippines and South Korea) and has demonstrated good reliability and validity, with Cronbach’s alphas ranging from 0.72 to 0.80 (Kim et al., 2005; Shim and Schwartz, 2008; Oei and Raylu, 2009; Magno, 2010; Oh and Lee, 2014). Examples of items in the questionnaire include: one should consider the needs of others before considering one’s own needs; one should not deviate from familial and social norms (Kim et al., 1999).

#### The parenting practices questionnaire

The Parenting Practices Questionnaire was a self-administered survey developed by Robinson et al. (1995) to assess parents’ parenting practices. The scale was with 62-items scored on a five-point rating scale (1 = never; 5 = always) to assess parents’ adherence to three major parenting styles: *authoritative (27 items)*, *authoritarian (20 items),* and *permissive (15 items)*. This questionnaire excluded items related to the rejecting/neglecting parenting style based on the assumption that participating parents consented to participate in the study willingly and were actively involved in their children’s activities (Robinson et al., 1995). The scale has been tested in various countries (Utah, Canada, Iran and Italy) with good reliability and validity (Robinson et al., 1996; Maria et al., 2013; Delvecchio et al., 2020; Mojdehi et al., 2020). Example items in the questionnaire include: “I praise my child when he/she is good”; “I argue with my child;” “I use threats such as punishment with little or no justification” (Robinson et al., 1995).

#### Pictorial scale of perceived competence and acceptance for young children preschool-kindergarten (male and female versions)

The PSPCSA was used to assess children’s perceived competence. Harter and Pike (1984) developed the 24-item scale with pictures covering the domains of cognitive competence (six items), physical competence (six items), peer acceptance (six items), and maternal acceptance (6 items). The scale has been tested in various countries showing good reliability and validity (Fantuzzo et al., 1996; Venetsano et al., 2018; Heritage et al., 2020). Each item has two pictures: one image represents a child who can perform the task, and the second image denotes a child who cannot perform the same task. Children are asked to indicate the picture that was the closest or most similar to them when performing the illustrated task. If the picture with a capable child is selected, the children are asked to indicate the extent of similarity between the two choices. Examples of items in the survey include: “This girl is not very good at counting. Are you?”; “The boy is not very good at climbing. Are you?” (Harter and Pike, 1984). Children are asked to make a judgment about the level of similarity between the illustrated picture and their self-perception on the same task.

### Procedures

The major caregiver of each child was invited to complete two sets of self-administered questionnaires. The children were interviewed in their classrooms during their free time by trained research assistants using the PSPCSA. The pictorial questionnaire for children took about 15 min to complete.

Before administering the questionnaires, participating parents and their children provided informed consent. The participants were informed about the study’s purpose, anonymity, and their right to withdraw from the study at any time. The completed questionnaire responses were entered into SPSS for analysis (Field, 2013).

### Data analysis

Responses to the survey items were coded such that higher scores reflected more positive responses to examine the relationships between Asian cultural values and parenting styles, relationships between Asian cultural values and children’s perceived competence, and the relationship between parenting style and children’s perceived competence. Multiple linear regression was found appropriate for this study as it allows relationships between variables to be constructed and explored (Draper and Smith, 1998).

## Results

### Reliabilities of scales

The reliabilities of the scales were good. For the Parenting Practices Questionnaire, the Cronbach’s alphas of authoritative, authoritarian, and permissive subscales in the parenting scale are 0.902, 0.743, and 0.616, respectively. For the Asian Values Scale, the Cronbach’s alphas for subscale family recognition through achievement, collectivism, and humility were 0.772, 0.688, and 0.649, respectively. The internal consistencies of subscale conformity to norms, emotional self-control and filial piety were low, with 0.454, 0.401, and 0.307, respectively. Therefore, these three subscales were removed in subsequent data analyzes. For PSPCSA, the Cronbach’s alphas of cognitive competence, physical competence, peer acceptance, maternal acceptance are 0.615, 0.448, 0.526, and 0.561, respectively.

### Intergroup differences in Asian cultural values and parenting style adoption

The study shows that South Asian parents maintained higher adherence to Asian cultural values (*R*^2^ = 0.597) than local Chinese, indicating a strong positive correlation with authoritative parenting style. The regression models of South Asian parents and the other two parenting styles, namely authoritarian (*R*^2^ = 0.145) and permissive (*R*^2^ = 0.268) failed to obtain satisfactory *R*^2^ scores, indicating the absence of a meaningful relationship between these two parenting styles and Asian cultural values. For local-Chinese parents, no regression models had higher *R*^2^ scores, as there were no meaningful relationships between any form of parenting style and their Asian cultural values. Regarding the influences of Asian cultural values on parenting style adoption, in South Asian parent group, authoritative parenting style had stronger positive correlation with *humility* (*β* = 0.490) than *collectivism* (*β* = 0.121). Authoritarian parenting style had negative correlation with c*ollectivism* (*β* = −0.205). Permissive parenting style had very weak negative correlations with the measured values including *achievement* (*β* = −0.134), humility (*β* = −0.027) and collectivism (*β* = −0.001). For local Chinese parents, authoritative parenting style had negative correlation with *achievement* (*β* = −0.316) whereas had the weakest correlation with *humility* (*β* = 0.013). Authoritarian parenting style had positive correlations with *achievement* (*β* = 0.315) and *collectivism* (*β* = 0.162). Permissive parenting style had weak correlations with measured values including a*chievement* (*β* = 0.266), humility (*β* = −0.143) and collectivism (*β* = 0.092) (Tables 1–3).

**Table 1:** Multiple linear regression of Asian cultural values adherence on parenting style for south Asians parents (*N* = 49).

Model	*B*	*SE*	*β*	*t*	*R* ^2^	*F*	*df*	*p*
Dependent variable: Parent – Authoritative					0.597	10.134	6	0.000
AVS–Conformity	0.179	0.121	0.168	1.481				0.146
AVS–Achievement	−0.010	0.055	−0.021	−0.179				0.859
AVS–Emotion	0.151	0.066	0.281	2.292				0.027
AVS–Collectivism	0.064	0.069	0.121	0.938				0.354
AVS–Humility	0.310	0.069	0.490	4.483				0.000
AVS–Filial Piety	−0.023	0.067	−0.038	−0.344				0.733
Dependent variable: Parent – Authoritarian					0.145	1.156	6	0.348
AVS–Conformity	0.037	0.173	0.035	0.212				0.833
AVS–Achievement	0.012	0.078	0.025	0.148				0.883
AVS–Emotion	−0.025	0.094	−0.047	−0.262				0.794
AVS – Collectivism	−0.107	0.098	−0.205	−1.088				0.283
AVS – Humility	0.097	0.099	0.157	0.982				0.332
AVS – Filial Piety	−0.226	0.095	−0.383	−2.380				0.002
Dependent variable: Parent – Permissive					0.268	2.498	6	0.037
AVS – Conformity	0.077	0.141	0.083	0.545				0.589
AVS – Achievement	−0.055	0.064	−0.134	−0.856				0.397
AVS – Emotion	−0.134	0.076	−0.291	−1.756				0.086
AVS – Collectivism	0.000	0.080	−0.001	−0.005				0.996
AVS – Humility	−0.015	0.080	−0.027	−0.184				0.855
AVS – Filial Piety	−0.235	0.077	−0.453	−3.038				0.004

AVS, Asian values scales.

**Table 2:** Multiple linear regression of Asian cultural values adherence on parenting style for local-Chinese parents (*N* = 48).

Model	*B*	*SE*	*β*	*t*	*R* ^2^	*F*	*df*	*p*
Dependent variable: Parent – Authoritative					0.275	2.596	6	0.032
AVS – Conformity	0.054	0.106	0.082	0.506				0.616
AVS – Achievement	−0.099	0.047	−0.316	−2.090				0.043
AVS – Emotion	0.090	0.073	0.190	1.236				0.223
AVS – Collectivism	0.035	0.076	0.068	0.463				0.646
AVS – Humility	0.010	0.110	0.013	0.089				0.930
AVS – Filial piety	0.127	0.101	0.196	1.253				0.217
Dependent variable: Parent – Authoritarian					0.17	1.398	6	0.239
AVS – Conformity	−0.033	0.122	−0.048	−0.273				0.786
AVS – Achievement	0.106	0.054	0.315	1.948				0.058
AVS – Emotion	−0.036	0.083	−0.071	−0.434				0.667
AVS – Collectivism	0.090	0.087	0.162	1.036				0.306
AVS – Humility	0.095	0.127	0.122	0.751				0.457
AVS – Filial piety	−0.059	0.116	−0.085	−0.508				0.614
Dependent variable: Parent – Permissive					0.125	0.973	6	0.456
AVS – Conformity	−0.101	0.104	−0.174	−0.973				0.336
AVS – Achievement	0.074	0.046	0.266	1.602				0.117
AVS – Emotion	0.059	0.071	0.142	0.838				0.407
AVS – Collectivism	0.042	0.074	0.092	0.575				0.569
AVS – Humility	−0.092	0.108	−0.143	−0.857				0.397
AVS – Filial piety	0.019	0.099	0.034	0.197				0.844

AVS, Asian values scales.

**Table 3:** Correlations between Asian values and parenting style.

Correlations								
Ethnic group			AVS - Family recognition through achievement	AVS - Collectivism	AVS - Humility	Parenting - Authoritative	Parenting - Authoritarian	Parenting - Permissive
South Asian	AVS - Family recognition through achievement	Pearson correlation	1	−0.278	−0.106	−0.135	−0.055	−0.204
		Sig. (2-tailed)		0.056	0.473	0.359	0.712	0.164
		*N*	48	48	48	48	48	48
	AVS - Collectivism	Pearson correlation	−0.278	1	0.342^*^	0.493^**^	−0.077	−0.003
		Sig. (2-tailed)	0.056		0.017	0.000	0.604	0.982
		*N*	48	48	48	48	48	48
	AVS - Humility	Pearson correlation	−0.106	0.342^*^	1	0.660^**^	0.078	−0.077
		Sig. (2-tailed)	0.473	0.017		0.000	0.594	0.599
		*N*	48	48	49	49	49	49
	Parenting - authoritative	Pearson correlation	−0.135	0.493^**^	0.660^**^	1	−0.005	−0.123
		Sig. (2-tailed)	0.359	0.000	0.000		0.973	0.400
		*N*	48	48	49	49	49	49
	Parenting - authoritarian	Pearson correlation	−0.055	−0.077	0.078	−0.005	1	0.588^**^
		Sig. (2-tailed)	0.712	0.604	0.594	0.973		0.000
		*N*	48	48	49	49	49	49
	Parenting - permissive	Pearson correlation	−0.204	−0.003	−0.077	−0.123	0.588^**^	1
		Sig. (2-tailed)	0.164	0.982	0.599	0.400	0.000	
		*N*	48	48	49	49	49	49
Local-Chinese	AVS - Family recognition through achievement	Pearson correlation	1	0.224	−0.260	−0.360^*^	0.340^*^	0.271
		Sig. (2-tailed)		0.125	0.074	0.012	0.018	0.062
		*N*	48	48	48	48	48	48
	AVS - Collectivism	Pearson correlation	0.224	1	0.208	0.039	0.240	0.102
		Sig. (2-tailed)	0.125		0.156	0.795	0.101	0.491
		*N*	48	48	48	48	48	48
	AVS - Humility	Pearson correlation	−0.260	0.208	1	0.237	0.018	−0.196
		Sig. (2-tailed)	0.074	0.156		0.104	0.903	0.182
		*N*	48	48	48	48	48	48
	Parenting - authoritative	Pearson correlation	−0.360^*^	0.039	0.237	1	−0.231	−0.213
		Sig. (2-tailed)	0.012	0.795	0.104		0.114	0.147
		*N*	48	48	48	48	48	48
	Parenting - authoritarian	Pearson correlation	0.340^*^	0.240	0.018	−0.231	1	0.287^*^
		Sig. (2-tailed)	0.018	0.101	0.903	0.114		0.048
		*N*	48	48	48	48	48	48
	Parenting - permissive	Pearson correlation	0.271	0.102	−0.196	−0.213	0.287^*^	1
		Sig. (2-tailed)	0.062	0.491	0.182	0.147	0.048	
		*N*	48	48	48	48	48	48

* Correlation is significant at the 0.05 level (2-tailed).

** Correlation is significant at the 0.01 level (2-tailed).

### Asian cultural values on children’s perceived competence

The correlation between Asian cultural values and perceived competence among children from local Chinese parents was significantly stronger than among South Asian parents. Specifically, the association between Asian cultural values and *peer* competence (*R^2^* = 0.643) was significantly stronger among local Chinese than their South Asian counterparts (*R^2^* = 0.097).

This study shows that all six domains of Asian cultural values were positively related to the four aspects (*cognitive*, *physical*, *peer*, and *maternal competence*) of children’s competence in both groups. For the local Chinese group, *collectivism* (*β* = 0.848) and *humility* (*β* = −0.838) have the strongest correlation with peer competence. A similar magnitude of these two values was also found in its strong correlation with *maternal* competence (*collectivism β* = 0.624; *humility β* = −0.758)*.* For the South Asian group, *cognitive competence* and *collectivism* (*β* = −0.324) had stronger correlations with the *physical* competence of children’s perceived competence (Tables 4–6).

**Table 4:** Correlations between Asian values and children competence.

Correlations												
Ethnic group			AVS - Conformity to norms	AVS - Family recognition through achievement	AVS - emotional self-control	AVS - Collectivism	AVS - Humility	AVS - Filial piety	C_Cognitive	C_Physical	C_Peer	C_Maternal
South Asian	AVS - Conformity to norms	Pearson correlation	1	0.311^*^	0.157	0.170	0.242	0.183	0.235	−0.008	0.122	0.018
		Sig. (2-tailed)		0.032	0.287	0.247	0.094	0.208	0.116	0.956	0.419	0.908
		*N*	49	48	48	48	49	49	46	46	46	46
	AVS - Family recognition through achievement	Pearson correlation	0.311^*^	1	−0.238	−0.278	−0.106	0.371^**^	0.250	0.359^*^	0.140	−0.052
		Sig. (2-tailed)	0.032		0.103	0.056	0.473	0.009	0.098	0.016	0.358	0.734
		*N*	48	48	48	48	48	48	45	45	45	45
	AVS - emotional self-control	Pearson correlation	0.157	−0.238	1	0.568^**^	0.297^*^	−0.174	−0.094	−0.046	−0.110	−0.036
		Sig. (2-tailed)	0.287	0.103		0.000	0.041	0.237	0.540	0.763	0.470	0.815
		*N*	48	48	48	48	48	48	45	45	45	45
	AVS - Collectivism	Pearson correlation	0.170	−0.278	0.568^**^	1	0.342^*^	−0.266	−0.302^*^	−0.198	−0.225	−0.093
		Sig. (2-tailed)	0.247	0.056	0.000		0.017	0.068	0.044	0.193	0.137	0.542
		*N*	48	48	48	48	48	48	45	45	45	45
	AVS - Humility	Pearson correlation	0.242	−0.106	0.297^*^	0.342^*^	1	−0.002	−0.106	0.007	−0.037	−0.301^*^
		Sig. (2-tailed)	0.094	0.473	0.041	0.017		0.988	0.485	0.964	0.809	0.042
		*N*	49	48	48	48	49	49	46	46	46	46
	AVS - Filial Piety	Pearson correlation	0.183	0.371^**^	−0.174	−0.266	−0.002	1	0.225	0.377^**^	0.220	0.052
		Sig. (2-tailed)	0.208	0.009	0.237	0.068	0.988		0.133	0.010	0.142	0.732
		*N*	49	48	48	48	49	49	46	46	46	46
	C_Cognitive	Pearson correlation	0.235	0.250	−0.094	−0.302^*^	−0.106	0.225	1	0.126	0.284	−0.007
		Sig. (2-tailed)	0.116	0.098	0.540	0.044	0.485	0.133		0.405	0.056	0.961
		*N*	46	45	45	45	46	46	46	46	46	46
	C_Physical	Pearson correlation	−0.008	0.359^*^	−0.046	−0.198	0.007	0.377^**^	0.126	1	−0.279	−0.042
		Sig. (2-tailed)	0.956	0.016	0.763	0.193	0.964	0.010	0.405		0.060	0.781
		*N*	46	45	45	45	46	46	46	46	46	46
	C_Peer	Pearson correlation	0.122	0.140	−0.110	−0.225	−0.037	0.220	0.284	−0.279	1	0.008
		Sig. (2-tailed)	0.419	0.358	0.470	0.137	0.809	0.142	0.056	0.060		0.955
		*N*	46	45	45	45	46	46	46	46	46	46
	C_Maternal	Pearson correlation	0.018	−0.052	−0.036	−0.093	−0.301^*^	0.052	−0.007	−0.042	0.008	1
		Sig. (2-tailed)	0.908	0.734	0.815	0.542	0.042	0.732	0.961	0.781	0.955	
		*N*	46	45	45	45	46	46	46	46	46	46
Local-Chinese	AVS - Conformity to norms	Pearson correlation	1	0.116	0.396^**^	0.220	0.268	0.424^**^	−0.041	−0.264	0.231	−0.170
		Sig. (2-tailed)		0.431	0.005	0.133	0.066	0.003	0.860	0.248	0.314	0.462
		*N*	48	48	48	48	48	48	21	21	21	21
	AVS - Family recognition through achievement	Pearson correlation	0.116	1	−0.193	0.224	−0.260	−0.145	−0.197	−0.166	0.094	−0.165
		Sig. (2-tailed)	0.431		0.190	0.125	0.074	0.325	0.391	0.471	0.685	0.476
		*N*	48	48	48	48	48	48	21	21	21	21
	AVS - Emotional self-control	Pearson correlation	0.396^**^	−0.193	1	0.134	0.236	0.354^*^	−0.093	0.120	0.300	0.038
		Sig. (2-tailed)	0.005	0.190		0.365	0.107	0.014	0.690	0.604	0.187	0.872
		*N*	48	48	48	48	48	48	21	21	21	21
	AVS - Collectivism	Pearson correlation	0.220	0.224	0.134	1	0.208	−0.023	0.127	0.131	0.433^*^	0.062
		Sig. (2-tailed)	0.133	0.125	0.365		0.156	0.876	0.585	0.572	0.050	0.789
		*N*	48	48	48	48	48	48	21	21	21	21
	AVS - Humility	Pearson correlation	0.268	−0.260	0.236	0.208	1	0.309^*^	0.480^*^	0.343	−0.330	−0.326
		Sig. (2-tailed)	0.066	0.074	0.107	0.156		0.033	0.028	0.128	0.143	0.150
		*N*	48	48	48	48	48	48	21	21	21	21
	AVS - Filial piety	Pearson correlation	0.424^**^	−0.145	0.354^*^	−0.023	0.309^*^	1	0.182	0.216	−0.120	−0.031
		Sig. (2-tailed)	0.003	0.325	0.014	0.876	0.033		0.429	0.348	0.604	0.894
		*N*	48	48	48	48	48	48	21	21	21	21
	C_Cognitive	Pearson correlation	−0.041	−0.197	−0.093	0.127	0.480^*^	0.182	1	0.406	−0.349	0.108
		Sig. (2-tailed)	0.860	0.391	0.690	0.585	0.028	0.429		0.068	0.121	0.642
		*N*	21	21	21	21	21	21	21	21	21	21
	C_Physical	Pearson correlation	−0.264	−0.166	0.120	0.131	0.343	0.216	0.406	1	−0.203	0.133
		Sig. (2-tailed)	0.248	0.471	0.604	0.572	0.128	0.348	0.068		0.377	0.565
		*N*	21	21	21	21	21	21	21	21	21	21
	C_Peer	Pearson correlation	0.231	0.094	0.300	0.433^*^	−0.330	−0.120	−0.349	−0.203	1	0.297
		Sig. (2-tailed)	0.314	0.685	0.187	0.050	0.143	0.604	0.121	0.377		0.191
		*N*	21	21	21	21	21	21	21	21	21	21
	C_Maternal	Pearson correlation	−0.170	−0.165	0.038	0.062	−0.326	−0.031	0.108	0.133	0.297	1
		Sig. (2-tailed)	0.462	0.476	0.872	0.789	0.150	0.894	0.642	0.565	0.191	
		*N*	21	21	21	21	21	21	21	21	21	21

* Correlation is significant at the 0.05 level (2-tailed).

** Correlation is significant at the 0.01 level (2-tailed).

**Table 5:** Multiple linear regression of Asian cultural values on children’s perceived competence for local Chinese children (*N* = 24).

Model	*B*	*SE*	*β*	*t*	*R* ^2^	*F*	*df*	*p*
Dependent variable: Children – Cognitive competence					0.285	0.822	6	0.571
AVS – Conformity	0.218	0.286	0.230	0.760				0.933
AVS – Achievement	−0.032	0.103	−0.103	−0.315				0.727
AVS – Emotion	−0.066	0.163	−0.103	−0.406				0.622
AVS – Collectivism	0.008	0.160	0.016	0.050				0.968
AVS – Humility	0.314	0.214	0.418	1.469				0.140
AVS – Filial Piety	0.077	0.189	0.108	0.407				0.833
Dependent variable: Children – Physical competence					0.229	1.014	6	0.455
AVS – Conformity	0.022	0.295	0.023	0.074				0.107
AVS – Achievement	−0.061	0.106	−0.195	−0.575				0.503
AVS – Emotion	−0.008	0.167	−0.013	−0.051				0.446
AVS – Collectivism	0.242	0.165	0.477	1.467				0.694
AVS – Humility	0.035	0.220	0.047	0.160				0.374
AVS – Filial piety	−0.026	0.195	−0.037	−0.136				0.202
Dependent variable: Children – Peer competence					0.643	3.865	6	0.017
AVS – Conformity	0.075	0.232	0.069	0.321				0.772
AVS – Achievement	−0.114	0.083	−0.315	−1.365				0.127
AVS – Emotion	−0.032	0.132	−0.043	−0.243				0.709
AVS – Collectivism	0.499	0.130	0.848	3.835				0.002
AVS – Humility	−0.720	0.173	−0.838	−4.162				0.003
AVS – Filial piety	0.110	0.154	0.134	0.714				0.663
Dependent variable: Children – Maternal competence					0.424	1.351	6	0.299
AVS – Conformity	−0.349	0.449	−0.211	−0.778				0.423
AVS – Achievement	−0.263	0.161	−0.476	−1.628				0.186
AVS – Emotion	−0.250	0.255	−0.223	−0.979				0.598
AVS – Collectivism	0.559	0.252	0.624	2.222				0.056
AVS – Humility	−0.994	0.335	−0.758	−2.968				0.022
AVS – Filial piety	0.128	0.297	0.103	0.430				0.851

AVS, Asian values scales.

**Table 6:** Multiple linear regression of Asian cultural values on children’s perceived competence for south Asian children (*N* = 81).

Model	*B*	*SE*	*β*	*t*	*R* ^2^	*F*	*df*	*p*
Dependent variable: Children – Cognitive competence					0.151	1.645	6	0.162
AVS – Conformity	0.218	0.194	0.191	1.123				0.085
AVS – Achievement	0.020	0.086	0.041	0.230				0.766
AVS – Emotion	−0.018	0.105	−0.031	−0.168				0.615
AVS – Collectivism	−0.138	0.108	−0.251	−1.277				0.78
AVS – Humility	−0.040	0.108	−0.062	−0.373				0.634
AVS – Filial piety	0.069	0.107	0.106	0.645				0.663
Dependent variable: Children – Physical competence					0.233	1.952	6	0.097
AVS – Conformity	−0.158	0.236	−0.108	−0.669				0.300
AVS – Achievement	0.139	0.104	0.222	1.328				0.061
AVS – Emotion	0.218	0.127	0.304	1.710				0.416
AVS – Collectivism	−0.229	0.132	−0.324	−1.735				0.581
AVS – Humility	−0.028	0.132	−0.034	−0.213				0.624
AVS – Filial piety	0.172	0.130	0.208	1.325				0.079
Dependent variable: Children – Peer competence					0.097	0.667	6	0.677
AVS – Conformity	0.130	0.211	0.108	0.616				0.451
AVS – Achievement	0.009	0.093	0.017	0.095				00.941
AVS – Emotion	0.038	0.114	0.065	0.337				0.986
AVS – Collectivism	−0.185	0.118	−0.319	−1.575				0.294
AVS – Humility	0.021	0.118	0.031	0.182				0.956
AVS – Filial piety	0.031	0.116	0.046	0.271				0.388
Dependent variable: Children – Maternal competence					0.125	0.864	6	0.530
AVS – Conformity	0.066	0.169	0.068	0.394				0.488
AVS – Achievement	−0.019	0.075	−0.046	−0.256				0.389
AVS – Emotion	0.073	0.091	0.154	0.808				0.787
AVS – Collectivism	−0.024	0.094	−0.051	−0.257				0.833
AVS – Humility	−0.207	0.094	−0.370	−2.203				0.047
AVS – Filial piety	0.006	0.093	0.010	0.061				0.623

AVS, Asian values scales.

### Relationship between parenting style and children’s perceived competence

The three parenting styles were not associated with children’s perceived competence in both groups. The regression models on parenting style and children’s perceived self-competence were low (*R*^2^ < 0.5; Table 7).

**Table 7:** Comparison of the first and second run of *R*^2^ multiple linear regression of parenting style affecting children’s perceived competence in local Chinese and south Asian groups.

	First run *R*^2^	Second run *R*^2^
Non-local Asian children		
Cognitive competence	0.018	0.028
Physical competence	0.06	0.054
Peer competence	0.058	0.059
Maternal competence	0.04	0.028
Local Chinese children		
Cognitive competence	0.202	0.069
Physical competence	0.222	0.132
Peer competence	0.118	0.062
Maternal competence	0.408	0.364

From the table above, all the parenting style models affecting children’s perceived competence were lower than 0.5, indicating the lack of a relationship between them. The models also showed that eliminating items with low correlation scores from the children’s perceived competence factors would not improve the regression results. Notably, during the second run, local Chinese children had an even lower *R*^2^, even though all *R*^2^ scores were less than 0.5.

## Discussion

### Intergroup differences in Asian cultural values and parenting style adoption

Authoritative parenting has long been associated with individualism dominating in the Western culture. It is considered the ideal parenting style with clear guidelines and a high level of responsiveness toward children’s needs (Huang and Gove, 2015). However, our results demonstrated that South Asian families with higher adherence to Asian culture values had greater authoritative parenting behaviors than their local counterparts. This contradictory finding against the traditional belief of “Asian–authoritarian parenting style” suggesting a need to re-examine the long-standing assumptions. Particularly, parents’ responsiveness to children’s needs in the Asian cultural context is needed to explore how parents apply Asian cultural values to pragmatically and meaningfully satisfy their children’s needs that determine parenting style rather than attributing the authoritarian parenting style to Asian cultural values.

#### South Asian parents

The relationship between Asian cultural values and parenting styles was evident in South Asian parents. However, it could not be discerned among the local Chinese parents, which could be explained by the goals and functional values of Asian cultural values among South Asian parents from an ecological perspective. Smooth transitions are required between various living contexts to promote a child’s well-being (Bronfenbrenner and Ceci, 1994). In this study, transition experiences between micro-and macro-context for ethnic minorities could be different from their local counterparts (Barker and Cornwell, 2019). Being ethnic minorities residing in Hong Kong, South Asian group would pay more emphasis on cultural accommodation than local Chinese group (Berry, 1980; Vu et al., 2019). With this, children being obedient and conforming to rules become particularly important to these families as these values could promote safety from a cultural clash with the mainstream and assume smoother adaptation to the host culture (Barker, 2015; Chan and Li, 2020). Hence, South Asian group would exercise the Asian cultural values of obedience and conformity as means to practice love and care to address children’s needs during the initial process of cultural accommodation and later acculturation (Bornstein, 2012; Stamkou et al., 2019; Chan and Li, 2020). Conversely, local Chinese do not have the pressure to accommodate to the host culture, and the functional values of Asian cultural values in meeting their children’s well-being are less incentivizing than their non-local counterparts. Finally, unlike the traditional beliefs of permissive indulgent parents who care about their children’s needs but have difficulty setting limits in children’s disciplines, the weak correlations Asian cultural values between and permissive parenting style in in both groups suggested that the characteristics of permissive parenting style had no alignment with Asian cultural values that are characterized with social order and hierarchy (Xu et al., 2005; Mojdehi et al., 2020).

#### Local Chinese parents

The relationship between authoritarian parenting style and the Asian cultural value of *achievement (family recognition through achievement)* was stronger than the other Asian cultural values in local Chinese parents, despite considerably less adhering to Asian cultural values, in our study. The finding suggested that the local Chinese parents may take authoritarian parenting behaviors as functional means to achieve their expectations on children’s achievement. The higher fidelity to the plans, the more control and monitoring would be needed, the more likely the expected outcomes can be achieved. If parents would like the child to obtain achievement to certain level, the closer the child follow their parents’ plans, the more likely the child can achieve the expected level of achievement (Huang and Gove, 2015).

Similarly, local Chinese parents with permissive parenting style demonstrated a comparably higher positive correlation with *achievement* than the other cultural values. This result suggested that parents with indulgent parenting behaviors have expectation in children’s achievement which implies these parents believe that indulgent parenting behaviors could be an optimal parenting style for their families and their children. A number of studies on indulgent parenting style suggested that its chances to bring positive developmental outcomes in children could be no less than authoritative (Chen et al., 2000; García and Gracia, 2014). Children raised with permissive parenting experienced less nervousness and could learn competent attitudes and behaviors from social behaviors of their parents (Garcia et al., 2021).

Regarding the role of *achievement* played in parents with authoritative parenting style, the moderate negative correlation between this style and *achievement* indicates that parents who expressed warmth and were sensitive to their children’s needs were less likely to consider *achievement* more important than other Asian cultural values such as *collectivism* and *humility*. These parents are could more timely knowledge and understanding about the priority needs at early years development. Moral and character development could be considered as more important values than achievement for parents with authoritative parenting styles (Huang and Gove, 2015; Hung, 2018).

### Components of Asian cultural values and parenting styles

A closer examination a closer examination of the association of specific components of Asian cultural values on parenting style shows that *collectivism* and *humility* contributed to authoritative parenting behaviors in South Asian families. From a cultural and pragmatic perspective, *collectivism* and *humility* are not merely values cherished by Asian families but are also recognized as protective factors for the survival and well-being of ethnic minorities in the local cultural context (Ang and Goh, 2006). In a collectivist society, a clear social order, interdependence, and mutual good are emphasized and considered essential for maintaining social harmony (Rudy and Grusec, 2001). Collectivism provides spiritual, physical, social, and emotional support to community members amid adversities, which is critical for ethnic minorities (Sorkhabi, 2005). Collectivistic behavior provides children with strong spiritual resources, protects them from harm, and promotes their well-being in the mainstream culture (Rudy and Grusec, 2001; Lansford, 2021). Moreover, parents with more humility than others are likely to recognize their limitations, become aware of their strengths, encourage their children to acknowledge their needs, and identify themselves without devaluing their home values. According to cultural studies on adaptation and well-being, forced assimilation can be detrimental to individual well-being as people who experience enforced cultural assimilation tend to devalue their cultural background and family values to integrate into the dominant culture (Berry, 1980; Liu et al., 2009; Barker, 2015). Such devaluation can be predominantly seen among children who have a high need for acceptance and peer relationships during childhood because children’s experiences and sense of achievement tend to be social, as opposed to adolescents who are academically oriented (Seligman, 2011; Barker, 2015). The perceived notion of the association between Asian cultural values and authoritarian parenting style or less desirable parenting practices must be re-visited and re-examined from the perspective of the significance of cultural values for the betterment of individuals’ well-being (Xu et al., 2005).

### Components of Asian cultural values and children’s perceived competence

This study found that all the three measured domains of Asian cultural values were positively related to all four aspects of children’s competence that were measured in this study. However, the specific Asian cultural values contributing to the specific aspects of competence varied between South Asian and local Chinese groups.

#### Collectivism

For the South Asian group, *collectivism* had negative correlations with *physical* competence, and *peer* competence, weak negative correlation with *cognitive* competence, and non-significant association with *maternal* competence. This suggests that the value of interdependency for *collectivism* could negatively affect children’s school-related competence. In contrast, *collectivism* had significantly stronger positive correlations with *physical* competence, *peer* competence, and *maternal* competence, and non-significant association with *cognitive* competence in the local Chinese group than their South Asian counterparts. Such findings can be explained by the functional meaning of collectivism in practice in these two groups. South Asian children are an ethnic minority in the local population. Group-oriented practices among members of a minority group may highlight their “differences” and pose an obstacle to their accommodation and integration process. Practicing group cohesiveness and prioritization of the group over the individual goals or needs may not be an optimal integration strategy for a minority group to adapt and integrate into the mainstream (Barker, 2015). Hence, to facilitate the accommodation and integration process, children of the minority group may tend to their needs to adapt to the host culture. Furthermore, they may develop a feeling of belonging to the mainstream group by being behaviorally and cognitively more similar to their mainstream peers.

In contrast, collectivism in the local Chinese group would positively contribute to children’s competence could be attributed to the identity of being a member of mainstream group (Rhee et al., 1995). Being mainstreamers would bring a strong sense of security to the communities. Mainstream values and practices are commonly considered the standard of societal expectations about how an individual should behave in society (Barker and Cornwell, 2019). Children with group-oriented identities and practices contribute to their competence of interacting with peers in everyday physical and cognitive interaction. The higher the group identity as mainstreamers, the more likely the individual would be accepted by the society, including his or her major caregivers (*maternal* competence in this study). When collectivism could lead to high social-related competence, including peer and maternal competence, group goal and achievement could be more important than individual achievement in the local Chinese group where mainstream values are exemplified as a group.

#### Achievement

South Asian and local Chinese groups could have different interpretations on *family recognition through achievement (achievement)*. For South Asian parents, *achievement* was associated with children’s *physical* competence but it had very weak associations with the rest of the children’s competence. Physical development and capabilities are considered as more important than socio-emotional related development in South Asian group which can be attributed to their disadvantaged living environment (Huang et al., 2012). The environment commonly associated with poor hygiene and crimes (Nguyet and Leung, 2014). Therefore, it is reasonable to assume these families are more likely to take pride if their children are able to grow up with both physical and mentally resilient strengths. On the other hand, for local Chinese group, *achievement* had negative relationships with *maternal* acceptance and *peer* competence than other competences suggested that local Chinese parents valued socio-emotional related competence more than achievement which traditionally referred to academic achievement. In recent years, the importance of holistic development in young children has been widely promoted in public education delivered through parent education programs at schools, community health clinics, and media channels (Ng et al., 2020). With more voices shared in public media asking for non-competitive curriculum and learning environment, local Chinese parents have developed high awareness about the non-academic needs in nurturing competent children (Ng et al., 2020).

#### Humility

For the South Asian group, *humility* had a moderate negative association with *maternal* competence and very weak associations with the rest of the measured children’s competence. On the contrary, *humility* was found to have very strong negative correlations with *peer* competence, *maternal* competence, and positive correlation with cognitive competence but had a weak correlation with *physical* competence in the local Chinse group. The phenomenon can be explained by the functional meaning of *humility* in these two groups. Humility in Confucian doctrine is interpreted as being humble, tolerant and unostentatious without boasting about one’s achievement (Shek, 2006; Chuang et al., 2018). For South Asian group, parents may take pride of their children’s achievement to promote the recognition by the mainstreamers and enhance the social status as member of ethnic minorities (Nguyet and Leung, 2014).

For both groups, the value of *humility* could impose different functions to the groups. For South Asian groups who are living in disadvantageous living environment, children being tolerate without showing off one’s strengths, both intellectual and physical strengths, could put their children at the risk of bullying (Nguyet and Leung, 2014). For ethnic minorities residing in a local population while socializing in more than one cultures (home and host), survival and adaptation in these social contexts are needed for smoother transition across contexts (Bronfenbrenner and Morris, 1998). It does not necessarily mean South Asian group does not value humility in raising their children. Rather, humility could be applied to various extent depending on the needs of these families in contexts (Nguyet and Leung, 2014). Similarly, the strong negative association between *humility*, children’s *peer* competence and *maternal* competence in local Chinese group does not necessarily mean local Chinese parents do not value humility in children nurturance. The traditional expectation for the humility in collectivist context may imply hindering one’s strengths and even uniqueness (Chan et al., 2009). A more adaptable application humility that value both humility and individual uniqueness would help strike the balance between the two emphasis. Such application would require the individuals to analyze and reflect (*cognitive* competence) on the needs and appropriateness in accordance to the social norm of their engaged context (Ang et al., 2007).

### Relationship between parenting style and children’s perceived competence

The null relationship between parenting style and children’s perceived competence suggests that parenting style does not lead to more competent children. Ideal parenting practices should be interpreted from within meaningful contexts. With reference to PPCT model, South Asian parents’ parenting practices that allow children to take pride in their homes’ cultural values and uniqueness while being aware of adjusting and adapting to their host culture, are likely to promote easier transition between social contexts with multiple norms (Bronfenbrenner and Morris, 1998). No individual is exposed solely to a single culture or norm. Social norms are dynamic, varying across social contexts and between people (Johnson and Puplampu, 2008). Parents must nurture children with skills and competencies to respond to the ever-changing social norms (Schwartz et al., 2010). Therefore, ideal parenting practices are behaviors responsive to changes that emerge in various contexts and enable children to be open and flexible to embrace diversity at home, in school, and within their community (Xu et al., 2005).

### Implications

The three important implications of this study include, contextualization of cultural values, the functional value of cultural norms in parenting practices, and reconceptualization of ideal parenting styles.

#### Contextualization of cultural values for Asian sub-cultural groups

*Asian cultural value* is a generic and inclusive term that homogenizes the complexity and intergroup diversity among various sub-cultural groups in any Asian city. The study’s findings provide deeper insights into typical Asian cultural values that have long been literally interpreted and associated with negative connotations in the Western literature, specifically in the context of involved culture and familial needs (Chan et al., 2009; Wong et al., 2018). The motives underlying values such as obedience, collectivism, and social hierarchy, exercised by South Asian parents, aim at smoother cultural accommodation and meaning of love, which is responsive to children’s needs rather than blindly exercising control over the dependents. Values in cultures have different meanings and perform varied functions for different goals to meet developmental needs in different immediate social contexts across one’s lifespan. Further research is required to examine the meaning, role, and functions of Asian cultural values at different stages of people’s lives in Asian sub-cultural groups.

#### Pragmatism of Asian cultural values for ethnic minorities

Cultural studies claim that an ideal integration is acculturation (Barker, 2015). Acculturation attenuates individuals’ pressure of adjusting to a new cultural environment and enables them to maintain and value their homes’ cultural values while positively integrating into the new culture such that smooth transition between social contexts can be promoted (Barker, 2015). A positive cultural integration process allows for maintaining cultural integrity alongside participation in the host cultural context (Yuen, 2016). In this study, the pragmatic value of collectivism and humility have higher tempting values for adoption and adherence for vulnerable people, i.e., those who are subject to social exclusion and are under pressure for social adaptation or acceptance. The study’s results can alert clinical researchers in family studies and family counseling practitioners to help ethnic minorities who blindly assimilate the host culture’s values. They should be helped in a caring and responsive way to increase their awareness of their needs and uniqueness. Parents of ethnic minorities should also be helped to nurture young children with attitudes, skills and resilient strength that enable them to face cultural clashes, be flexible and open to multi-cultural or norm acculturation in the broader cultural context is essential for the well-being of all community members, both ethnic minorities and local population (Stamkou et al., 2019).

#### Rethinking ideal parenting

Ideal or appropriate parenting, regardless of the types of parenting styles, is determined by identifying if parenting practices can foster children’s well-being, including addressing the needs of both family and children in meaningful contexts and promoting smooth transition across daily living contexts (Darling and Steinberg, 2017). The study reiterates to clinical researchers in family studies and family counseling practitioners that authoritarian parenting style, as well as other so called less ideal parenting styles, is not necessarily associated with negative outcomes in children. In addition, awareness and competence in applying culturally sensitive intervention strategies are critical to improving service quality and appropriateness for families from diverse backgrounds.

### Limitations

This study has several limitations. First, the questionnaires for adherence to Asian cultural values and parenting practices were self-reported, and participating parents rated their parenting style based on the host cultural norms. Second, due to the caregivers in families with lower socioeconomic status are mothers, the gender of caregivers has not been investigated in this study. With the unitary gender type of the participating caretakers, the gender of the child was not included in this study as the exploration on dynamic between both genders of caretakers and children could be very limited. Third, this study did not investigate the relationships between indulgent-neglectful parenting styles on child development as parents with this parenting style paid the least effort to understand children’s needs and set limits in children’s behaviors. Investigations on the influences of these parents on their children development could be very limited. Fourth, the difference in the number of children in South Asian and local Chinese group could post limitation on findings generalization. Finally, although existing cultural research suggested that Asian cultural values and practices have been highly influenced by Confucianism, Taoism and Buddhism (Vuong et al., 2018, 2020), this study did not explore the relationships between these doctrines and the values held by the members of each participating ethnic group for the following reason. Apart from local Chinese, since the South Asian participants in this study were collectively called “South Asian” without differentiating each ethnic group due to limited sample size with uneven ethnic group distribution and limited statistical power, the investigation on the influences of the three doctrines on participants’ cultural values and parenting behaviors could be very limited.

Considering the above limitations, future research is suggested to explore the role of Asian cultural values adherence in fathers on parenting style and children’s development. Also, in view of diverse ethnicities among Asian, particularly South Asian, population, future research could enhance the statistical power by expanding sample size with more balanced ethnic make up in the sample and investigating the relationship between each specific component of cultural values, including the three doctrines, parenting behaviors in each ethnic group with its unique socio-cultural history to explore the influences of cultural values in parenting practices of different Asian ethnic groups on children development. After all, although the present study with collected quantitative data can be improved and supplemented by interviewing parents, conducting on-site observations of children’s social behaviors, expanding the sample size of both South Asian and local Chinese group, and enhancing the differentiation of different cultural groups among the participants, our results have invaluable reference values and provide pioneer insights in exploring contextualization of parenting styles for different ethnic groups within a geographical region rather than across countries and valuable reference for future research on parenting amid cultural diversities.

## Conclusion

Globalization facilitates cultural mobility within and across regions and countries. Parenting in multicultural societies is challenging and must be responsive to the changing dynamics in children’s socio-cultural contexts that enable them to live with competence and dignity. This study complements the literature on parenting young children from diverse Asian sub-cultural groups. Clinical researchers in family studies and family counseling practitioners could use these findings to gain insight into the existing influences of home and host culture on parenting practices. Further, they could use these findings to integrate cultural sensitivity into family interventions by contextualizing cultural values and interpreting them as pragmatic parenting practices for families living in metropolitan cities, such as Hong Kong. Longitudinal research could explore the impact of specific parenting behaviors on local and immigrant children’s development and well-being at different life stages.

## Data availability statement

The original contributions presented in the study are included in the article/supplementary material, further inquiries can be directed to the corresponding author.

## Ethics statement

The studies involving human participants were reviewed and approved by Hong Kong Metropolitan University. Written informed consent to participate in this study was provided by the participants' legal guardian/next of kin.

## Author contributions

The author confirms being the sole contributor of this work and has approved it for publication.

## Conflict of interest

The author declares that the research was conducted in the absence of any commercial or financial relationships that could be construed as a potential conflict of interest.

## Publisher’s note

All claims expressed in this article are solely those of the authors and do not necessarily represent those of their affiliated organizations, or those of the publisher, the editors and the reviewers. Any product that may be evaluated in this article, or claim that may be made by its manufacturer, is not guaranteed or endorsed by the publisher.
